# Prepregnancy Prediabetes and Large‐for‐Gestational‐Age Birth Independent of Gestational Diabetes Among Asian Women

**DOI:** 10.1111/jog.70548

**Published:** 2026-10-26

**Authors:** Tatsuo Inamura, Sho Tano, Sumire Fujito, Kazuya Fuma, Seiko Matsuo, Yuki Nozaki, Satoru Katsuki, Masato Yoshihara, Shigeru Yoshida, Mamoru Yamashita, Hiroaki Kajiyama, Takafumi Ushida

**Affiliations:** ^1^ Department of Obstetrics and Gynecology Nagoya University Graduate School of Medicine Nagoya Japan; ^2^ Kishokai Medical Corporation Inazawa Japan

**Keywords:** gestational diabetes mellitus, large for gestational age, preconception care, prediabetes, pregnancy

## Abstract

**Aim:**

Prediabetes may increase the likelihood of adverse perinatal outcomes, but evidence regarding prepregnancy prediabetes remains limited. This study examined its associations with gestational diabetes mellitus (GDM) and the delivery of a large‐for‐gestational‐age (LGA) infant.

**Methods:**

This multicenter retrospective study included women who delivered at 12 obstetric facilities in Japan between January 1, 2014, and December 31, 2024, and had undergone hemoglobin A1c (HbA1c) measurement within 1 year before conception. The primary maternal and fetal outcomes were GDM and LGA birth, respectively. Multivariable logistic regression analyses were performed to evaluate each outcome, with adjustment for maternal age, assisted reproductive technology, prepregnancy body mass index, and primiparity.

**Results:**

A total of 282 pregnancies were included, of which 19 (6.7%) were complicated by prepregnancy prediabetes. Prepregnancy prediabetes showed a more than twofold increase in the estimated odds of GDM (adjusted odds ratio [aOR], 2.15; 95% confidence interval [CI], 0.53–8.66), although the association was not statistically significant. In contrast, it was significantly associated with the delivery of an LGA infant (aOR, 3.48; 95% CI, 1.14–10.66). After women who developed GDM were excluded, 262 women remained, including 16 (6.1%) with prepregnancy prediabetes. In this subgroup, prepregnancy prediabetes remained significantly associated with the delivery of an LGA infant (aOR, 3.49; 95% CI, 1.09–11.57).

**Conclusion:**

Prepregnancy prediabetes was independently associated with higher odds of delivering an LGA infant among Asian women, including those without a diagnosis of GDM. These findings suggest that preconception dysglycemia may help identify women at increased risk of excessive fetal growth beyond GDM diagnosis and warrant further evaluation in larger studies.

## 1 Introduction

Prediabetes has emerged as a potential risk factor for adverse pregnancy and perinatal outcomes, including gestational diabetes mellitus (GDM) and excessive fetal growth [1, 2, 3, 4, 5, 6]. However, evidence regarding the association of prepregnancy prediabetes with these outcomes remains limited, particularly among Asian populations. To date, a large US multiethnic preconception cohort has examined the association between prepregnancy prediabetes and perinatal outcomes [2]; to our knowledge, no comparable study has been conducted in an Asian population. Compared with Western populations, Asian populations tend to develop glucose intolerance at lower body mass index (BMI) values and exhibit greater impairment of insulin secretion [7, 8, 9]; therefore, findings from Western populations may not be directly generalizable to Asian women.

Although the identification of prepregnancy prediabetes may be clinically important, routine hemoglobin A1c (HbA1c) testing before conception is not generally feasible in obstetric care settings. Women diagnosed with GDM receive dietary counseling, glucose monitoring, and pharmacological treatment when necessary [10]. However, if women with prepregnancy prediabetes are not diagnosed with GDM, they may not receive any targeted intervention despite remaining at increased risk of adverse perinatal outcomes. Accordingly, two key clinically important questions remain: whether prepregnancy prediabetes is associated with adverse perinatal outcomes among Asian women and whether routine GDM screening during pregnancy identifies women with prepregnancy prediabetes as having GDM.

Among perinatal outcomes, large‐for‐gestational‐age (LGA) birth is of particular clinical importance because it is associated with neonatal intensive care unit (NICU) admission, respiratory morbidity, neonatal hypoglycemia, birth trauma, stillbirth, and neonatal death [11, 12], as well as increased long‐term risks of obesity and metabolic syndrome in the offspring [13, 14]. Therefore, this study investigated the associations of prepregnancy prediabetes with GDM diagnosed during pregnancy and the delivery of an LGA infant in an Asian population and determined whether women with prepregnancy prediabetes were identified as having GDM during pregnancy through routine screening.

## 2 Methods

### 2.1 Study Design

This multicenter retrospective study used electronic medical records from pregnant women aged ≥ 18 years who delivered at 12 obstetric facilities operated by Kishokai Medical Corporation located in the Tokai region of Japan between January 1, 2014, and December 31, 2024, and who had undergone HbA1c measurement within 1 year before conception. HbA1c levels were measured as part of routine outpatient care. To ensure data quality, records containing maternal weight values < 30 or > 150 kg were considered implausible and excluded from the analysis [15]. Women were excluded if they had prepregnancy diabetes mellitus, defined as an HbA1c level ≥ 6.5% measured in the nonpregnant state within 1 year before conception; a miscarriage before 22 weeks of gestation; or missing data on prepregnancy BMI (Figure 1).

**FIGURE 1:**
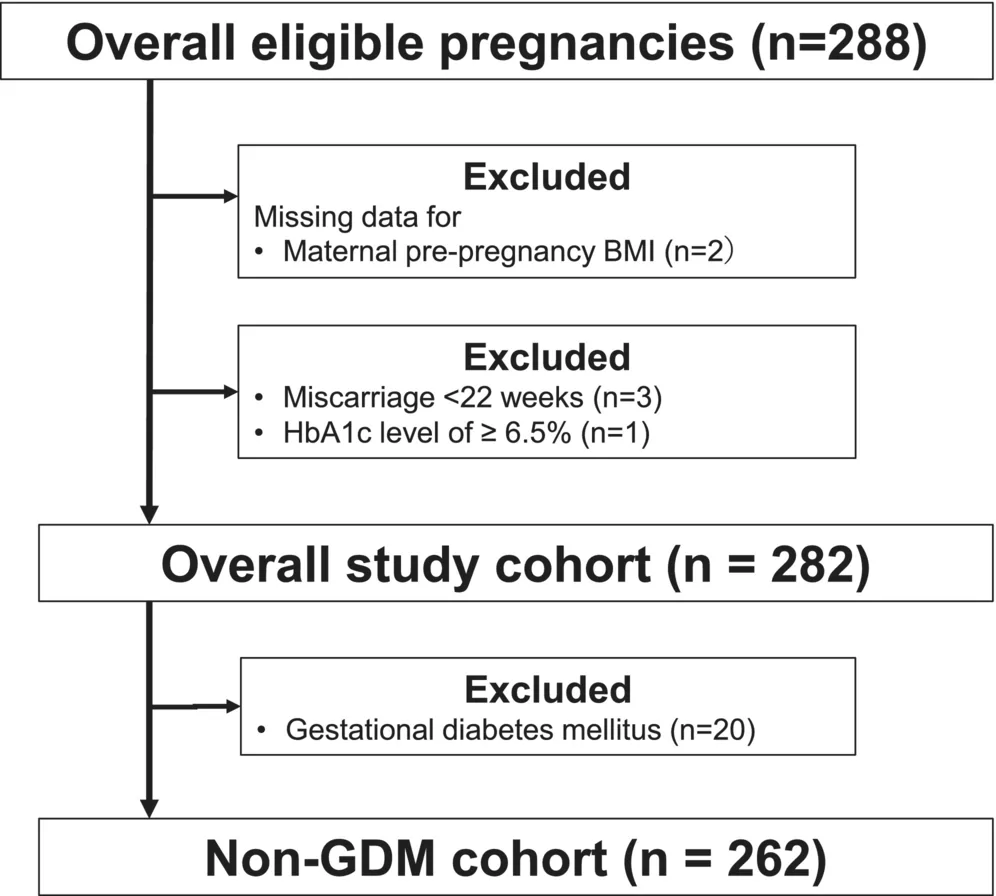
Flowchart of the study population. Of the 288 eligible pregnancies, exclusions were made for missing maternal prepregnancy BMI, miscarriage before 22 weeks of gestation, and prepregnancy diabetes mellitus. The study cohort consisted of 282 pregnancies. After excluding 20 women with GDM, 262 pregnancies were included in the non‐GDM cohort. BMI, body mass index; GDM, gestational diabetes mellitus; HbA1c, hemoglobin A1c.

### 2.2 Ethics Approval

This study was approved by the Ethics Committee of Nagoya University Hospital (approval no. 2015–0415) and was conducted in accordance with the Declaration of Helsinki. The ethics committee waived the requirement to obtain informed consent because of the retrospective study design.

### 2.3 Definitions of Variables

Prepregnancy prediabetes was defined as an HbA1c level of 5.7%–6.4%, measured in the nonpregnant state within 1 year before conception. Normoglycemia was defined as an HbA1c level < 5.7% [16, 17].

GDM was diagnosed during routine clinical practice according to Japanese guidelines [10]. Women with a screen‐positive random blood glucose result (≥ 100 mg/dL [5.6 mmol/L]) during early pregnancy or mid‐pregnancy or a positive 50‐g glucose challenge test (1‐h blood glucose level ≥ 140 mg/dL [7.8 mmol/L]) underwent a 75‐g oral glucose tolerance test (OGTT). GDM was diagnosed when at least one of the following International Association of Diabetes and Pregnancy Study Groups thresholds was met: fasting blood glucose ≥ 92 mg/dL (5.1 mmol/L), 1 h blood glucose ≥ 180 mg/dL (10.0 mmol/L), or 2 h blood glucose ≥ 153 mg/dL (8.5 mmol/L). Infants were classified as small‐for‐gestational‐age (SGA) or LGA when their birth weights were below the 10th percentile or at or above the 90th percentile for gestational age, respectively, according to Japanese birth weight standards [18, 19].

Self‐reported maternal height and prepregnancy body weight were used to calculate prepregnancy BMI (kg/m^2^). The calculated BMI values were categorized as underweight (< 18.5 kg/m^2^), normal weight (18.5–24.9 kg/m^2^), or overweight (≥ 25.0 kg/m^2^) [20]. Assisted reproductive technology (ART) was defined as conception achieved through in vitro fertilization or intracytoplasmic sperm injection. Hypertensive disorders of pregnancy (HDP) were defined as new‐onset hypertension after 20 weeks of gestation, with a systolic blood pressure ≥ 140 mmHg or diastolic blood pressure ≥ 90 mmHg [21].

### 2.4 Outcomes

The co‐primary outcomes were GDM diagnosed during pregnancy as the maternal outcome and the delivery of an LGA infant as the fetal outcome.

### 2.5 Statistical Analysis

Statistical analyses were performed using IBM SPSS Statistics, version 31.0.1.0 (IBM Corp., Armonk, NY, USA), and Python version 3.11.9 (https://www.python.org/). Continuous variables are presented as means ± standard deviations (SDs) and categorical variables as numbers and percentages. Statistical significance was defined as a two‐sided *p*‐value < 0.05. Crude odds ratios (cORs) and adjusted odds ratios (aORs), with their corresponding 95% confidence intervals (CIs), were estimated for the co‐primary outcomes using univariable and multivariable logistic regression analyses, respectively. The models for LGA birth and GDM included maternal age, use of ART, prepregnancy BMI, and primiparity, which were selected as covariates based on previous studies [21, 22, 23, 24, 25, 26, 27, 28, 29, 30, 31, 32, 33]. GDM was additionally included as a covariate in the models for LGA birth. Two multivariable models were constructed to assess the robustness of the associations to alternative specifications of prepregnancy BMI. In Model 1, prepregnancy BMI was entered as a continuous variable, whereas in Model 2, it was entered as a categorical variable.

### 2.6 Use of Artificial Intelligence

ChatGPT (OpenAI) and DeepL (DeepL SE) were used for translation and academic language editing during the preparation of this manuscript. After using these tools, the authors reviewed and revised all tool‐assisted outputs as required and take full responsibility for the accuracy, integrity, and content of the final manuscript.

## 3 Results

### 3.1 Characteristics of Study Cohort

A total of 282 pregnancies were included, of which 19 (6.7%) occurred in women with prepregnancy prediabetes. The maternal characteristics and perinatal outcomes of the normoglycemic and prediabetes groups are shown in Table 1. The mean prepregnancy HbA1c levels were 5.2% ± 0.2% and 5.8% ± 0.1%, respectively. The mean interval between HbA1c measurement and conception did not differ significantly between the groups (23.4 ± 15.0 vs. 29.4 ± 13.2 weeks, *p* = 0.069). The incidence of LGA birth was significantly higher in the prediabetes group than in the normoglycemic group (LGA, 31.6% vs. 13.4%, *p* = 0.042), whereas the incidence of GDM was numerically, but not significantly, higher in the prediabetes group (15.8% vs. 6.5%, *p* = 0.142).

**TABLE 1:** Baseline characteristics and pregnancy outcomes according to prepregnancy prediabetes status in the overall cohort.

	Normoglycemia, *n* = 263	Prediabetes, *n* = 19	*p*
Maternal age, years	31.9 ± 4.3	30.0 ± 4.6	0.097
Primiparity	142 (54.0)	5 (26.3)	0.036
Assisted reproductive technology	21 (8.0)	1 (5.3)	1.000
Prepregnancy body mass index, kg/m^2^	22.0 ± 3.6	24.0 ± 3.8	0.040
Underweight	36 (13.7)	0 (0.0)	0.146
Normal weight	176 (66.9)	14 (73.7)	0.723
Overweight	51 (19.4)	5 (26.3)	0.550
Prepregnancy HbA1c, %	5.2 ± 0.2	5.8 ± 0.1	—
Timing of HbA1c measurement before pregnancy, weeks	23.4 ± 15.0	29.4 ± 13.2	0.069
Hypertensive disorders of pregnancy	16 (6.1)	1 (5.3)	1.000
Gestational diabetes mellitus	17 (6.5)	3 (15.8)	0.142
GDM at early pregnancy	5 (1.9)	1 (5.3)	—
GDM at mid‐pregnancy	12 (4.6)	2 (10.5)	—
Gestational age at delivery, weeks	39.4 ± 1.6	39.2 ± 1.4	0.557
Preterm birth (< 37 weeks)	10 (3.8)	0 (0.0)	—
Birth weight, g	3066.3 ± 395.8	3165.1 ± 475.4	0.387
Low birth weight (birth weight < 2500 g)	11 (4.2)	1 (5.3)	0.574
SGA	19 (7.3)	1 (5.3)	1.000
LGA	35 (13.4)	6 (31.6)	0.042
Placental weight, g	598.7 ± 358.9	606.7 ± 115.3	0.817
Female infant	126 (47.9)	6 (31.6)	0.254
GDM screening positive at early pregnancy	18 (7.2)	3 (18.8)	0.120
GDM screening positive at middle pregnancy	40 (15.9)	6 (37.5)	0.038
Emergency Cesarean section	30 (11.4)	4 (21.1)	0.657
Stillbirth at ≥ 22 weeks of gestation	1 (0.4)	0 (0.0)	—

*Note:* Data are presented as mean ± standard deviation, *n* (%).

Abbreviations: GDM, gestational diabetes mellitus; LGA, large for gestational age; SGA, small for gestational age.

### 3.2 Association Between Prepregnancy Prediabetes and GDM

In the multivariable analysis of GDM (Table 2), prepregnancy prediabetes was not significantly associated with GDM in Model 1 (aOR, 2.15; 95% CI, 0.53–8.66). ART was also not significantly associated with GDM (aOR, 1.66; 95% CI, 0.34–8.04). Similar results were obtained in Model 2, in which prepregnancy BMI was analyzed as a categorical variable; no significant association was observed between prepregnancy prediabetes and GDM (aOR, 2.38; 95% CI, 0.59–9.63).

**TABLE 2:** Logistic regression analysis for GDM.

	Model 1		Model 2	
	cOR (95% CI)	aOR (95% CI)	cOR (95% CI)	aOR (95% CI)
Maternal age, years	1.00 (0.90–1.11)	1.00 (0.90–1.11)	1.00 (0.90–1.11)	1.00 (0.90–1.12)
Primiparity	0.59 (0.23–1.49)	0.63 (0.24–1.64)	0.59 (0.23–1.49)	0.63 (0.24–1.64)
ART	1.34 (0.29–6.21)	1.66 (0.34–8.04)	1.34 (0.29–6.21)	1.61 (0.33–7.75)
Prepregnancy BMI, kg/m^2^	1.08 (0.97–1.21)	1.08 (0.96–1.21)	—	—
Underweight	—	—	0.75 (0.17–3.35)	1.03 (0.21–4.99)
Overweight	—	—	1.82 (0.67–4.96)	1.79 (0.63–5.07)
Prediabetes	2.71 (0.72–10.23)	2.15 (0.53–8.66)	2.71 (0.72–10.23)	2.38 (0.59–9.63)

*Note:* Model 1 included prepregnancy BMI as a continuous variable; Model 2 included prepregnancy BMI as a categorical variable.

Abbreviations: aOR, adjusted odds ratio; ART, assisted reproductive technology; BMI, body mass index; CI, confidence interval; cOR, crude odds ratio.

### 3.3 Association Between Prepregnancy Prediabetes and LGA Birth

In the multivariable analysis of LGA birth (Table 3), prepregnancy prediabetes was significantly associated with higher odds of delivering an LGA infant in Model 1 (aOR, 3.48; 95% CI, 1.14–10.66). ART was also significantly associated with higher odds of delivering an LGA infant (aOR, 5.05; 95% CI, 1.91–13.30). GDM was not significantly associated with LGA birth (aOR, 1.23; 95% CI, 0.36–4.19). Similar results were obtained in Model 2, in which prepregnancy BMI was analyzed as a categorical variable (Table 3).

**TABLE 3:** Logistic regression analysis for LGA in the overall cohort.

	Model 1		Model 2	
	cOR (95% CI)	aOR (95% CI)	cOR (95% CI)	aOR (95% CI)
Maternal age, years	1.05 (0.97–1.13)	1.05 (0.97–1.14)	1.05 (0.97–1.13)	1.04 (0.96–1.14)
Primiparity	1.22 (0.62–2.37)	1.32 (0.64–2.72)	1.22 (0.62–2.37)	1.33 (0.65–2.74)
ART	4.91 (1.94–12.41)	5.05 (1.91–13.3)	4.91 (1.94–12.41)	4.80 (1.81–12.90)
Prepregnancy BMI, kg/m^2^	1.07 (0.98–1.16)	1.08 (0.98–1.18)	—	—
Underweight	—	—	0.16 (0.02–1.20)	0.20 (0.03–1.52)
Overweight	—	—	1.35 (0.63–2.91)	1.51 (0.67–3.37)
Prediabetes	2.99 (1.07–8.39)	3.48 (1.14–10.66)	2.99 (1.07–8.39)	3.42 (1.11–10.26)
GDM	1.51 (0.48–4.78)	1.23 (0.36–4.19)	1.51 (0.48–4.78)	1.25 (0.37–4.21)

*Note:* Model 1 included prepregnancy BMI as a continuous variable; Model 2 included prepregnancy BMI as a categorical variable.

Abbreviations: aOR, adjusted odds ratio; ART, assisted reproductive technology; BMI, body mass index; CI, confidence interval; cOR, crude odds ratio; GDM, gestational diabetes mellitus.

### 3.4 Association Between Prepregnancy Prediabetes and LGA Birth Among Women Without GDM

After excluding women with GDM, 262 pregnancies remained, 16 (6.1%) of which occurred in women with prepregnancy prediabetes. Maternal characteristics and pregnancy outcomes according to prepregnancy glycemic status are presented in Supplemental Table 1. The incidence of LGA birth was higher among women with prepregnancy prediabetes than among those with normoglycemia (31.2% vs. 13.1%); however, the unadjusted between‐group difference did not reach statistical significance (*p* = 0.059).

In the multivariable logistic regression analysis of LGA birth (Table 4), prepregnancy prediabetes remained associated with higher odds of delivering an LGA infant after adjustment for maternal age, primiparity, prepregnancy BMI, and ART (Model 1: aOR, 3.49; 95% CI, 1.09–11.57). This association remained significant when prepregnancy BMI was analyzed as a categorical variable (Model 2: aOR, 3.36; 95% CI, 1.02–10.96).

**TABLE 4:** Logistic regression analysis for LGA among women without GDM.

	Model 1		Model 2	
	cOR (95% CI)	aOR (95% CI)	cOR (95% CI)	aOR (95% CI)
Maternal age, years	1.05 (0.97–1.14)	1.05 (0.96–1.14)	1.05 (0.97–1.14)	1.05 (0.96–1.14)
Primiparity	1.20 (0.60–2.42)	1.32 (0.63–2.75)	1.20 (0.60–2.42)	1.32 (0.63–2.78)
ART	3.79 (1.40–10.25)	3.78 (1.34–10.69)	3.79 (1.40–10.25)	3.62 (1.27–10.32)
Prepregnancy BMI, kg/m^2^	1.08 (0.98–1.18)	1.08 (0.99–1.20)	—	—
Underweight	—	—	0.18 (0.02–1.34)	0.21 (0.03–1.66)
Overweight	—	—	1.45 (0.65–3.26)	1.61 (0.70–3.70)
Prediabetes	3.03 (0.99–9.28)	3.49 (1.09–11.57)	3.03 (0.99–9.28)	3.36 (1.02–10.96)

*Note:* Model 1 included prepregnancy BMI as a continuous variable; Model 2 included prepregnancy BMI as a categorical variable.

Abbreviations: aOR, adjusted odds ratio; ART, assisted reproductive technology; BMI, body mass index; CI, confidence interval; cOR, crude odds ratio; GDM, gestational diabetes mellitus.

## 4 Discussion

### 4.1 Principal Findings

In this multicenter study of Asian women, prepregnancy prediabetes was associated with approximately threefold higher odds of delivering an LGA infant after adjustment for maternal age, parity, prepregnancy BMI, and ART. Importantly, this association remained significant in the analysis restricted to women without GDM. In contrast, the point estimate suggested that prepregnancy prediabetes was associated with more than twofold higher odds of GDM diagnosed during pregnancy, although the association did not reach statistical significance. These findings suggest that prepregnancy prediabetes may identify an increased likelihood of excessive fetal growth that is not fully reflected by a diagnosis of GDM during pregnancy. Consequently, some women with prepregnancy prediabetes may remain unrecognized as having preconception dysglycemia during pregnancy and may not receive targeted metabolic interventions, despite having higher odds of LGA birth.

### 4.2 Comparison With Previous Studies

Previous studies conducted predominantly in Western populations have reported associations between prepregnancy or early‐pregnancy prediabetes and GDM, HDP, preterm birth, and excessive fetal growth [1, 2, 3, 4, 5, 6]. Our study extends these findings to an Asian population by showing that prepregnancy prediabetes was associated with approximately threefold higher adjusted odds of delivering an LGA infant. This association persisted after adjustment for prepregnancy BMI, including when BMI was analyzed as a categorical variable, suggesting that the association was not fully accounted for by maternal adiposity as measured by BMI. This finding may be particularly relevant to Asian populations, in whom glucose intolerance can develop at relatively low BMI levels, a tendency that has been attributed partly to greater impairment of insulin secretion [7, 8, 9].

The association remained significant after excluding women with GDM, suggesting that the observed association between prepregnancy prediabetes and LGA birth was also present among women without GDM. Prepregnancy HbA1c levels may therefore reflect metabolic dysfunction present before conception that is not necessarily detected by routine GDM screening. These findings indicate that some Asian women with preconception dysglycemia may not be identified as having abnormal glucose metabolism during pregnancy, despite having higher odds of delivering an LGA infant.

### 4.3 Interpretation of the Findings

Prepregnancy prediabetes was associated with more than twofold higher odds of GDM diagnosed during pregnancy, but this association did not reach statistical significance. A large US multiethnic preconception cohort reported a similarly increased risk of subsequent GDM among women with prediabetes (aRR, 2.21; 95% CI, 1.91–2.56) [2], consistent in magnitude with our estimate (aOR, 2.15; 95% CI, 0.53–8.66), although the association did not reach statistical significance in our smaller cohort. Therefore, the absence of statistical significance should not be interpreted as evidence of no association between prepregnancy prediabetes and GDM diagnosed during pregnancy.

Notably, the association between prepregnancy prediabetes and the delivery of an LGA infant remained significant after women diagnosed with GDM were excluded. This suggests that the higher odds of excessive fetal growth were not confined to women with GDM. Mild dysglycemia below the diagnostic threshold for GDM may increase transplacental glucose transfer and stimulate fetal insulin secretion, thereby promoting fetal growth [1]. Alternatively, a prepregnancy HbA1c level in the prediabetic range may reflect an underlying metabolic dysfunction that is not adequately captured by glucose testing performed at discrete time points during pregnancy.

Although these findings should be interpreted cautiously given the small sample and selectively tested sample, they support the possibility that women with prepregnancy prediabetes may have persistent dysglycemia during pregnancy despite not meeting the diagnostic criteria for GDM.

Although GDM is an established risk factor for LGA birth [34], GDM was not significantly associated with LGA birth in the present cohort. This finding should be interpreted cautiously because of the limited number of GDM cases and the wide CI. Management initiated after GDM diagnosis may also have attenuated the observed association with LGA [35, 36]; however, detailed treatment information was unavailable, and this possibility could not be formally evaluated. The persistence of the association between prepregnancy prediabetes and LGA among women without diagnosed GDM suggests that glycemic abnormalities below the diagnostic threshold for GDM may also contribute to excessive fetal growth.

### 4.4 Clinical Implications

Notably, only 3 of the 19 women with prepregnancy prediabetes (15.8%) were diagnosed with GDM during pregnancy. Thus, most women with prepregnancy prediabetes did not meet the diagnostic criteria for GDM, although prepregnancy prediabetes was associated with higher odds of delivering an LGA infant. Because routine HbA1c assessment before conception is uncommon, many women may enter pregnancy without their preconception glycemic status being recognized. These findings suggest that a diagnosis of GDM based on routine screening may not identify all women with prepregnancy dysglycemia who have higher odds of excessive fetal growth. Consequently, some of these women may not receive targeted dietary counseling, glucose monitoring, or other metabolic interventions.

Although these findings do not support universal preconception HbA1c screening, greater attention could be given to existing prepregnancy HbA1c results, particularly among women undergoing infertility evaluation or treatment. Preventing or managing prediabetes may also represent an important component of preconception care. Because increases in BMI over time have been associated with incident prediabetes in women [17], maintaining a healthy weight trajectory before pregnancy may help reduce preconception dysglycemia and the associated likelihood of excessive fetal growth.

For women with documented prepregnancy prediabetes, additional individualized metabolic assessment during pregnancy may merit consideration, even when routine GDM screening results are negative. Pregnancy may also provide an opportunity to recognize women at elevated long‐term metabolic risk and initiate preventive care, particularly because the progression from prediabetes to Type 2 diabetes may occur more rapidly in Asian populations than in Western populations [37, 38]. Such an approach could potentially improve both perinatal outcomes and long‐term maternal metabolic health. Prospective studies are needed to determine whether preconception risk management, enhanced glucose monitoring, or early lifestyle interventions can reduce the incidence of LGA birth and progression to Type 2 diabetes.

### 4.5 Strengths and Limitations

First, a major strength of this study was the assessment of prediabetes using an HbA1c measurement obtained before conception, allowing maternal glycemic status to be evaluated before pregnancy‐related metabolic changes occurred. The multicenter design and use of standardized Japanese diagnostic and classification criteria for GDM and fetal growth also enhanced the consistency and clinical applicability of these findings. In addition, the subgroup analysis excluding women who developed GDM enabled us to assess whether the association between prepregnancy prediabetes and LGA birth persisted among women without a GDM diagnosis.

This study has some limitations. First, its retrospective design precluded causal inference. Second, the sample size was limited, with relatively few GDM events and small numbers of women with prepregnancy prediabetes or ART pregnancies. Therefore, the multivariable estimates, particularly for GDM, may be unstable and should be interpreted cautiously. Third, HbA1c levels were not routinely measured in all pregnant women. Therefore, the participants constituted a selected population of women who had undergone HbA1c testing, most commonly during infertility‐related outpatient care; this selection may have introduced bias and may limit the generalizability of the findings to broader obstetric populations. Fourth, the timing of HbA1c measurement varied within 1 year before conception, and glycemic status could have changed between measurement and conception. Nevertheless, the mean interval was approximately 6 months, substantially shorter than the interval of more than 12 months reported in a previous study [2] potentially providing a more representative assessment of glycemic status near conception. Fifth, OGTT data were available only for women with positive screening results, limiting our ability to evaluate subthreshold glucose abnormalities among screen‐negative women. Sixth, information on fresh versus frozen–thawed embryo transfer was unavailable. Because frozen embryo transfer has been associated with LGA birth [26, 39], the strong ART–LGA association may partly reflect embryo transfer type or the small number of ART pregnancies. Finally, residual confounding by unmeasured factors, such as gestational weight gain, diet, physical activity, family history of diabetes, and socioeconomic status, could not be excluded.

In conclusion, prepregnancy prediabetes was associated with higher odds of delivering an LGA infant among Asian women, including those without a diagnosis of GDM. These findings highlight that preconception dysglycemia may be associated with excessive fetal growth even in the absence of GDM. Greater attention to documented prepregnancy glycemic status may help identify women at increased risk of LGA who would not otherwise be identified on the basis of a GDM diagnosis. Given the limited sample size and number of outcome events, larger prospective studies are needed to confirm these findings and evaluate appropriate surveillance or intervention strategies.

## Author Contributions

**Tatsuo Inamura:** data curation, formal analysis, investigation, methodology, writing – original draft, writing – review and editing. **Sho Tano:** conceptualization, formal analysis, methodology, investigation, funding acquisition, writing – original draft, writing – review and editing. **Sumire Fujito:** data curation, writing – review and editing, investigation. **Kazuya Fuma:** investigation, writing – review and editing. **Seiko Matsuo:** investigation, writing – review and editing. **Yuki Nozaki:** investigation, writing – review and editing. **Satoru Katsuki:** investigation, writing – review and editing. **Masato Yoshihara:** investigation, writing – review and editing. **Shigeru Yoshida:** data curation, writing – review and editing, investigation. **Mamoru Yamashita:** data curation, investigation, writing – review and editing. **Hiroaki Kajiyama:** writing – review and editing, supervision. **Takafumi Ushida:** investigation, supervision, writing – review and editing.

## Funding

This work was supported by a Grant‐in‐Aid for Scientific Research from the Japan Society for the Promotion of Science (grant no. 25K21070 to Sho Tano). This funding also supported the article processing charge. The funder had no role in the study design, data collection, analysis, or interpretation, manuscript preparation, or the decision to submit the article for publication.

## Disclosure

Hiroaki Kajiyama and Sho Tano are editorial board members of the journal and the co‐authors of this article. They were excluded from the peer‐review process and all editorial decisions related to the acceptance and publication of this article. Peer review was handled independently by the JOGR Journal editorial office to minimize bias.

## Ethics Statement

This study was approved by the Institutional Review Board of Nagoya University Hospital (approval number: 2015–0415). The study was conducted in accordance with the Declaration of Helsinki.

## Consent

The requirement for written informed consent was waived because of the retrospective nature of the study, and participants were provided with the opportunity to opt out in accordance with the approval of the institutional ethics committee.

## Conflicts of Interest

The authors declare no conflicts of interest.

## Supporting information


**Supplemental Table 1.** Baseline characteristics and pregnancy outcomes after excluding GDM.

## Data Availability

The data that support the findings of this study are not publicly available because they contain clinical information obtained from medical records and their public sharing was not included in the approval granted by the institutional ethics committee. The study was conducted under conditions that restrict the use of the data to the approved research purposes in order to protect participant confidentiality and privacy.
