# Supplementary material for: Prepregnancy Prediabetes and Large‐for‐Gestational‐Age Birth Independent of Gestational Diabetes Among Asian Women
Source: J Obstet Gynaecol Res. 2026 Oct 26;52(11):e70548. doi: 10.1111/jog.70548 (PMC13709328; doi:10.1111/jog.70548)
Supplement: Supplementary file 1 [file JOG-52-0-s001.docx]

**Supplemental Table 1. Baseline characteristics and pregnancy outcomes after excluding GDM.**

| Variable | Overall (n=262) | Normoglycemia (n=246) | Prediabetes (n=16) | *p* value |
| --- | --- | --- | --- | --- |
| Maternal age, years | 31.8 ± 4.4 | 31.9 ± 4.3 | 30.2 ± 4.9 | 0.197 |
| Primiparity | 139 (53.1) | 134 (54.5) | 5 (31.2) | 0.122 |
| ART | 20 (7.6) | 19 (7.7) | 1 (6.2) | 1 |
| Pre-pregnancy BMI, kg/m² | 22.0 ± 3.6 | 22.0 ± 3.6 | 23.0 ± 2.9 | 0.187 |
| Underweight | 34 (13.0) | 34 (13.8) | 0 (0.0) | 0.239 |
| Normal weight | 178 (67.9) | 165 (67.1) | 13 (81.2) | 0.368 |
| Overweight | 50 (19.1) | 47 (19.1) | 3 (18.8) | 1 |
| Pre-pregnancy HbA1c, % | 5.2 ± 0.2 | 5.2 ± 0.2 | 5.8 ± 0.2 | - |
| Timing of HbA1c measurement before pregnancy, weeks | 23.5 ± 15.1 | 23.1 ± 15.1 | 29.7 ± 14.2 | 0.09 |
| HDP | 15 (5.7) | 14 (5.7) | 1 (6.2) | 1 |
| Gestational age at delivery, weeks | 39.4 ± 1.6 | 39.4 ± 1.6 | 39.2 ± 1.5 | 0.545 |
| Preterm birth | 9 (3.4) | 9 (3.7) | 0 (0.0) | 1 |
| Birth weight, g | 3072.6 ± 402.2 | 3065.6 ± 396.8 | 3180.6 ± 479.6 | 0.361 |
| Low birth weight | 11 (4.2) | 10 (4.1) | 1 (6.2) | 0.507 |
| Macrosomia | 2 (0.8) | 1 (0.4) | 1 (6.2) | 0.119 |
| SGA | 20 (7.7) | 19 (7.8) | 1 (6.2) | 1 |
| LGA | 37 (14.2) | 32 (13.1) | 5 (31.2) | 0.059 |
| Placental weight, g | 600.2 ± 359.9 | 600.6 ± 370.4 | 595.5 ± 120.8 | 0.896 |
| Female infant | 120 (45.8) | 115 (46.7) | 5 (31.2) | 0.344 |
| Emergency Cesarean section | 32 (12.2) | 28 (11.4) | 4 (25.0) | 0.592 |
| Stillbirth at ≥22 weeks of gestation | 1 (0.4) | 1 (0.4) | 0 (0.0) | - |

Data are presented as mean ± standard deviation, n (%).

BMI, body mass index; ART, assisted reproductive technology; HDP, hypertensive disorders of pregnancy; GDM, gestational diabetes mellitus; GA, gestational age; SGA, small for gestational age; LGA, large for gestational age.
